# Determination of Volatile Organic Compounds and Antibacterial Activity of the Amazonian Cyanobacterium *Synechococcus* sp. Strain GFB01

**DOI:** 10.3390/molecules25204744

**Published:** 2020-10-16

**Authors:** Samuel Cavalcante do Amaral, Agenor Valadares Santos, Maria Paula da Cruz Schneider, Joyce Kelly Rosário da Silva, Luciana Pereira Xavier

**Affiliations:** 1 Laboratory of Biotechnology of Enzymes and Biotransformation, Biological Sciences Institute, Federal University of Para, Belém 66075-110, Brazil; samuel.amaral@icb.ufpa.br (S.C.d.A.); valadaresantos@gmail.com (A.V.S.); 2 Center of Genomics and Systems Biology, Biological Sciences Institute, Federal University of Para, Belém 66075-110, Brazil; mariapaulacruzschneider@gmail.com

**Keywords:** cyanobacteria, *Synechococcus* sp., volatiles organic compounds, 6-pentadecanol, octadecyl acetate

## Abstract

Cyanobacteria exhibit great biotechnological potential due to their capacity to produce compounds with various applicability. Volatile organic compounds (VOCs) possess low molecular weight and high vapor pressure. Many volatiles produced by microorganisms have biotechnological potential, including antimicrobial activity. This study aimed to investigate the VOCs synthesized by cyanobacterium *Synechococcus* sp. strain GFB01, and the influence of nitrate and phosphate on its antibacterial potential. The strain was isolated from the surface of the freshwater lagoon Lagoa dos Índios, Amapá state, in Northern Brazil. After cultivation, the VOCs were extracted by a simultaneous distillation-extraction process, using a Likens-Nickerson apparatus (2 h), and then identified by GC-MS. The extracts did not display inhibitory activity against the Gram-positive bacteria tested by the disk-diffusion agar method. However, the anti-*Salmonella* property in both extracts (methanol and aqueous) was detected. The main VOCs identified were heptadecane (81.32%) and octadecyl acetate (11.71%). To the best of our knowledge, this is the first study of VOCs emitted by a cyanobacterium from the Amazon that reports the occurrence of 6-pentadecanol and octadecyl acetate in cyanobacteria.

## 1. Introduction

Cyanobacteria are the most ancient group of photosynthetic bacteria of earth, and its presence contributed to the development of aerobic atmospheric in which allowed the appearance of new forms of life [1]. Moreover, it is known that they gave rise to the higher plant chloroplasts. Plant and cyanobacteria, therefore, possess several similarities [2]. These bacteria belong to a very diverse group distributed in several types of environments, including hot spring, cold deserts, oceans, hypersaline water, and freshwater [3,4]. The capacity to survive in various distinct habitats is due to its adaptive mechanisms, such as their ability to produce secondary metabolites with a large assortment of functions and structures [5,6]. In the literature, a considerable number of molecules identified in cyanobacteria with potential applications in pharmaceutical, food, and cosmetic industries, as well as biofuel and agriculture, have been described [7].

The quantity of bacteria resistant to antibiotics has expressively increased, leading to great concern among the members of the scientific community [8,9]. In this context, many types of research have been developed in order to discover new antibiotic compounds from distinct sources [10,11,12]. Cyanobacteria are considered to be a promising source of antibacterial compounds whose mechanism of action is very variable [13,14]. Many studies have mainly focused only on the evaluation of the activity of extracts, and there are few investigations to analyze the influence of nutrient input on the antagonist activity against pathogenic bacteria [15,16,17]. Phosphate and nitrate are essential nutrients in cyanobacteria metabolism. Their concentrations in the culture medium substantially affect the growth and the secondary metabolite profile of cyanobacteria [18,19].

Recently, a category of compounds produced by cyanobacteria has gained attention: the volatile organic compounds (VOCs) [20]. These compounds are produced and released into the environment by other types of bacteria, fungi, and plants. They are known for usually having a low molecular weight and high vapor pressure. Thus, they can quickly evaporate and migrate to the atmosphere [21]. The function of VOCs in cyanobacteria is still poorly understood. However, different biological roles are attributed to them, such as protection against bacterivores or other cyanobacteria, homeostasis and maintenance, preventing dehydration, cold-stress response, and signaling [22]. VOCs found in cyanobacteria can be classified into several chemical classes, such as terpenoids, fatty acid derivatives, amino acid derivatives, carbohydrate derivatives, and nor-terpenoids pathway products [23].

Geosmin and 2-methylborneol are some of the most frequent volatile compounds produced by cyanobacteria, which are “off-flavor” compounds associated with fouling taste and odor problems in drinking water and fish [24,25]. Cyanobacteria are a rich source of volatile metabolites that can be applied in several fields. Some of these compounds have shown antibacterial, anticancer, anti-cyanobacterial, antifungal, antioxidant, and anti-inflammatory activity [26,27,28,29,30]. In addition, cyanobacteria are capable of producing alkanes and alkenes by using fatty acid as substrates. Hydrocarbons and other volatiles compounds can be applied in the production of biofuel and fragrances, respectively [31,32].

**Figure 1:**
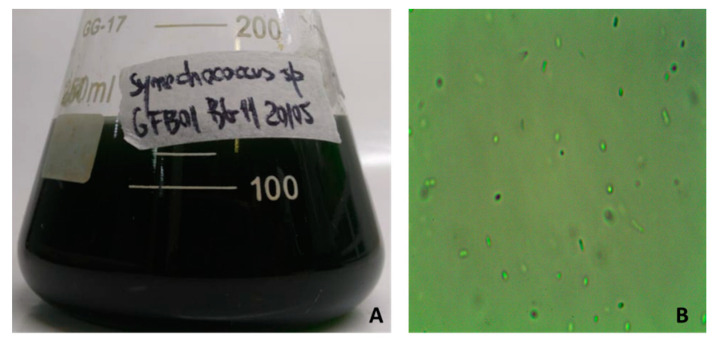
*Synechococcus* sp. GFB01 in BG11 medium (**A**) and visualized by optical microscope (**B**). Magnification: 1000× (Nikon H550L)**.**

The availability of nitrogen, carbon, sulfur, and phosphorus, as well as various environmental factors, such as illumination, pH value, temperature, and growth phase, can influence the chemical composition of cyanobacteria [33]. A study of cyanobacterium collected from a peculiar environment of the Amazon can provide the identification of unfamiliar VOCs and their biological potential. Thus, this work aimed at the identification and evaluation of the biotechnological potential of VOCs synthesized by freshwater cyanobacterium *Synechococcus* sp. strain GFB01 (Figure 1) from the Amazon region (Brazil). *Synechococcus* species are described as fast-growing unicellular organisms that are very abundant in the ocean and extremely relevant to the primary biomass production [4,34]. Due to their amenability to genetic manipulation, they have been used as a model organism to investigate pathways involved in the synthesis of secondary metabolites, as well as cell factories to produce commercially relevant molecules [35,36].

## 2. Results

**Figure 2:**
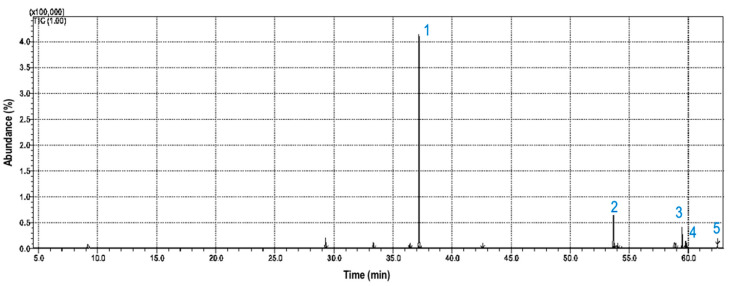
Ion chromatogram of GC-MS analysis of volatile fraction of *Synechococcus* sp. strain GFB01: (**1**) heptadecane, (**2**) octadecyl acetate, (**3**) pentadecane, (**4**) hexadecane, and (**5**) 6-pentadecanol.

Figure 2 represents the chromatogram obtained by GC-MS analysis of the volatile fraction of *Synechococcus* sp. GFB01. Heptadecane was the main compound identified, followed by octadecyl acetate (Table 1).

**Figure 3:**
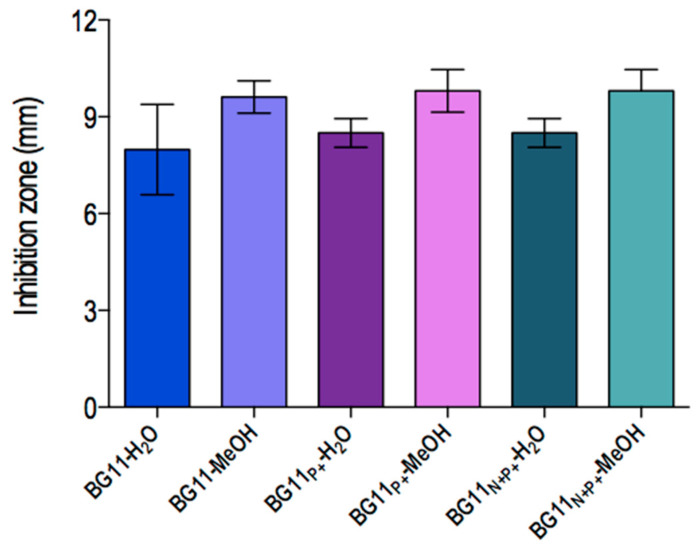
Antibacterial activity of *Synechococcus* sp. strain GFB01 extracts obtained from culture in different phosphate and nitrate concentrations against the Gram-negative bacterium *Salmonella typhimurium* ATCC 14021. The paper disk diameter was included.

Regarding the antibacterial assay, the extracts did not display inhibitory activity against the Gram-positive bacteria here tested. However, *Salmonella typhimurium* ATCC 14021 (American type culture collection, Rockville, MD, USA) had its growth inhibited by all samples except for extract BG11N+ varying from 7.6 ± 0.9 to 9.8 ± 0.6 mm. Lipophilic extracts exhibited higher antibacterial effects in comparison to hydrophilic extract. Nonetheless, these differences between the groups were not statically significant. Disks treated only with 0.1 M Tris-HCl buffer pH 7.1 or methanol (100%) did not show any antagonist activity (Figure 3).

## 3. Discussion

Heptadecane was the most abundant in the volatile fraction of *Synechococcus* sp. strain GFB01, and branched-chain alkanes were absent. Previous studies reported a strong relation between alkane chain length and cyanobacterial natural habitat. Heptadecane and pentadecane are related as the main compounds in cyanobacteria from freshwater and marine environments, respectively [37]. Moreover, studies suggested a relationship between morphological aspects and hydrocarbons’ profile to cyanobacteria. Branched-chain alkanes were detected only in filamentous cyanobacteria but very rare in unicellular strain. This distinct characteristic can be attributed to the fatty acid utilized as a precursor in the alkane synthesis or to the substrate specificity of enzymes involved in the addition of branches [38].

Cyanobacteria are one of the most important producers of alkanes in the ocean [39]. The genera *Prochlorococcus* and *Synechococcus* are estimated to release between 308 and 771 million tons of hydrocarbons per year [39]. Oil-degrading bacteria are capable of using these hydrocarbons and converting them to carbon dioxide, playing an essential role in the maintenance of these microorganisms [40]. The physiological role of hydrocarbons in cyanobacteria is unclear. However, in specific environmental conditions, the production of individual alkanes is enhanced [41].

*Synechocystis* sp. PCC 6803 strain cultivated at 20 °C displayed a substantial increase of heptadecane, which plays an essential role in cold tolerance [41]. In lagoon Lagoa dos Índios, the average temperature is between 23 and 30 °C [42]. This alteration may cause modifications in cell metabolism, including the heptadecane production level in *Synechococcus* sp. GFB01. The concentration of hydrocarbons in cyanobacteria is predominantly in the thylakoid and cytoplasmic membranes. In both layers, they are responsible for increasing flexibility and facilitating the curvature during cell growth. Non-hydrocarbon-producing genetically manipulated cyanobacteria exhibited a larger size when compared to the wild type. The absence of hydrocarbon also negatively affected cell growth and caused cell damage [43].

There are two known biosynthetic pathways for the production of hydrocarbon in cyanobacteria, which have never been detected simultaneously in the same organism [31]. Fatty acids are used as a common substrate for both pathways: One pathway is predominant and characterized by the conversion of fatty acyl-ACP to fatty acyl aldehyde and its posterior transformation into alkane by enzymes acyl-acyl carrier protein reductase (AAR) and aldehyde reformulating oxygenase (ADO), respectively [31,44]. The second pathway origins the α-olefins (OLS) by a large multidomain protein with homology to type I polyketide synthase [45,46]. The non-coexistence of the FAAR/ADO and OLS pathway suggests the action of selective pressure, which preserved a route while excluding others [31]. The chemical profile of VOCs identified in this study is associated with the AAR/ADO pathway, since heptadecane and pentadecane are found exclusively in cyanobacteria encoding these enzymes [31].

Alkanes here identified are very useful in the chemical industry as major constituents of various fuels, such as diesel, jet flew, and gasoline [47]. The VOCS produced by cyanobacterium *Spirulina platensis* showed antibacterial activity against Gram-positive and Gram-negative bacteria. Its major constituent was heptadecane [48]. Moreover, this compound exhibits the capacity of downregulating some metabolites related to the inflammatory process by activation of NF-kappaB transcription factor which persistently occurs in a considerable number of diseases, such as cancer, neurodegenerative disease, and asthma [30].

Octadecyl acetate was identified in other organisms, such as moths and bees, in their extracts of the sex pheromone gland and Dufour’s gland, respectively [49,50,51]. In addition, this component is present in the defensive secretion with repellent propriety of certain arthropods [52,53,54]. Moreover, in the females of *Eupoecilia ambiguella* (grape moth pest), it is responsible for increasing the capacity of attracting males when combined with (*Z*)-9-dodecenyl acetate and dodecyl acetate [52]. Besides, octadecyl acetate has been reported in the leaves, roots, and flowers of plants with medicinal and commercial value, including strawberries, galanga, rough lemon, potatoes, and pequi [55,56,57,58,59]. *Dracocephalum moldavica*, a medicinal plant whose essential oil has antiseptic and antibacterial properties, emitted this volatile after the exposure to salt stress, suggesting a protective role [60,61]. In the industry, the octadecyl acetate is very useful to produce shampoo, deodorants, candles, hairspray cream, and rinse, due to its low toxicity and lack of allergic response [62,63].

An unidentified C-15 aliphatic alcohol was found in a cyanobacterial mat by Rejmankova and co-workers [64]. This compound behaved as an attractive biological agent for *Anopheles* mosquitoes. The lagoon Lagoa dos Índios is regionally known as a hangover area characterized by a complex and peculiar environment with a great variety of species. Many organisms utilize this ecosystem only for shelter and breeding. Its natural conditions are favorable for the development of various species of Culicidae, mainly immature mosquitoes belonging to the genus *Anopheles* [65]. Based on the above-cited study, there is a possibility that 6-pentadecanol contributes to the high incidence of the *Anopheles*, including some vector of diseases, such as malaria, in this region. Further research will be relevant to better clarify this issue, and it may contribute to the prevention and control of these mosquitoes.

The chemical compound 6-pentadecanol is a fatty alcohol, a compound class that contains a significant number of applications in the cosmetic, pharmaceutical, and chemical industries [66]. Due to its lubricating, emollient, solubilizing, or emulsifying properties, these compounds can be used as biofuel additives [67]. In addition, long-chain aliphatic alcohols, comprising between six and twenty-two carbons atoms, exhibit low toxicity [68]. Some fatty alcohols showed inhibition of the growth of follicular bacterium *Propionibacterium acnes* responsible for inducing acne and inflammation [69]. The presence of hydrophilic and hydrophobic regions in these molecules contributes to its antimicrobial activity. Moreover, they have been used as a precursor of bioactive molecules such as pachastrissamine and *Commiphoru mukul* (guggul), which present cytotoxicity against human lung carcinoma cells and anti-inflammatory properties, respectively [70,71].

Cyanobacteria exhibit a high ability to produce important fatty alcohols through the use of metabolic engineering due to its photosynthetic capability and for being genetically manipulable [72,73]. Moreover, advances in DNA sequencing technology have favored the deposit of various cyanobacterial genomes in a short period and have allowed a greater understanding of their genetic systems [74]. The production of 6-pentadecanol in this cyanobacterium suggested the existence of an alcohol dehydrogenase able to reduce fatty aldehyde intermediate to fatty alcohol. NCBI search reveled a medium chain zinc-binding alcohol dehydrogenase family protein in the genome of *Synechococcus* sp. GFB01 (accession number: WP_048017391). Homologous protein was found by BLAST-protein on NCBI. This enzyme exhibits high similarity with other zinc-binding alcohol dehydrogenase belonged not solely to cyanobacteria, but also to other microorganisms. Zinc-binding alcohol dehydrogenase family requires zinc atom(s) as cofactor and can be encountered in mammals, plants, fungi, and bacteria [75]. They accomplish an enormous variety of functions in cell metabolism, including ethanol generation by *Saccharomyces cerevisiae* [76].

Some cyanobacteria isolated from Amazon have been considered as a promising source of biodiesel due to their fatty acid profile [77,78]. The alcohol 6-pentadecanol was identified in lesser quantity than heptadecane, which leads to inferring that acyl-ACP reductase action converted the most of fatty aldehydes generated to alkanes. There is little knowledge about alcohol dehydrogenases produced by cyanobacteria. However, its activity is induced by stress conditions [79]. Biochemical characterization of WP_048017391 would reveal its substrate affinity and also whether it preferably reduces aldehyde or oxidize alcohols. The cyanobacterium *Synechococcus elongatus* PCC 7942 was capable of producing fatty alcohol to generate wax ester. The incorporated biosynthetic pathway was formed by the co-expression of long-chain alcohol dehydrogenase and an acyl-ACP reductase from *Synechocystis* sp. PCC 6803, along with diacylglycerol acyltransferase [80].

The investigation of the antibacterial activity of cyanobacteria is mainly focused on filamentous species. However, few studies have been conducted on the bactericidal property of species from Chroococcales group in which include the genera *Synechocystis* and *Synechococcus* [81]. These compounds play a crucial ecological role by preventing the colonization of pathogenic bacteria in the mucilaginous sheath [14]. Gram-positive bacteria are usually the main target of antibiotic compounds from cyanobacteria [82,83]. In our study, the extracts of *Synechococcus* sp. GFB01 did not display inhibition against any Gram-positive bacteria. Therefore, these results indicate a different action mechanism in comparison to other substances found in these microorganisms.

Gram-negative bacteria have greater resistance to antibiotics due to their outer membrane composed of lipopolysaccharide (LPS), which impedes the entrance of these compounds inside the cell [82]. This membrane acts as a selective barrier where small hydrophilic antibiotics utilize the pore-forming porins to access the cell interior while hydrophobic molecules diffuse across the lipid bilayer [83]. The anti-*Salmonella* activity was detected in both aqueous and methanolic extract. Methanol is a solvent widely utilized during bioactive compounds extraction due to its amphiphilic nature, as well as being capable of dissolving non-polar compounds. Thus, there is the possibility of inhibitory compound with distinct action mechanism in *Synechococcus* sp. strain GFB01.

Comprehending factors involved in the production of bioactive compounds can provide insight into their functions in the cell and help optimize production for a particular substance commercially interesting. Nitrate and phosphate are essential elements for all types of cells, and few studies reported the effect of their concentrations on cyanobacterial antibiotic compounds [17]. In the study with *Synechococcus* sp. GFB01, the increase of nitrate concentration suppressed the antibacterial effect against *Salmonella typhimurium* ATCC 14021. It is known that phosphate is involved in the biosynthesis of many different types of antibiotics and a more significant number of other secondary metabolites, being recognized by its suppressor role [84]. In contrast, in this study, the supplementation with this element did not affect the inhibitory property of cyanobacterium. However, it was capable of reversing the suppressor role caused by enrichment with nitrate.

## 4. Materials and Methods

### 4.1. Strain and Culture Conditions

The cyanobacterium *Synechococcus* sp. GFB01 was selected from the culture collection of Center of Genomics and Systems Biology at Biological Sciences Institute, Federal University of Pará (Belém, Brazil). The strain was collected in 2011 from the surface of the freshwater lagoon Lagoa dos Índios, Macapá, Amapá State, Northern Brazil (0°01′55.1″ N 51°06′09.6″ W). This strain was the first of this genus isolated from South America to have its genome sequenced [85]. Cyanobacteria were grown photoautotrophically at a constant temperature of 25 °C, with controlled light conditions (12 h of light and 12 h of darkness) in BG-11 medium [86]. Modified BG11 medium was utilized to investigate the nitrate and phosphate influence on antibacterial property of *Synechococcus* sp. GFB01. In the first medium was added two-fold nitrate concentration (3.0 g/L) (BG11_N+_), while the second was supplemented with three-fold phosphate concentration (0.12 g/L) (BG11_P+_). The third medium showed both nitrate and phosphate concentration previously described (BG11_N+P+_).

### 4.2. Biomass Harvesting

The cyanobacterial cells were harvested from 1 L culture by centrifugation at 6000× *g* for 25 min. The supernatant was recovered and filtrated and tested for antibacterial activity, while the wet biomass was distributed in falcon-tube and frozen at −20 °C, until use.

### 4.3. Cyanobacterial Extract

The supernatant was frozen at −80 °C, freeze-dried, and then stored at −20 °C. The lyophilized material was resuspended in 0.1 M Tris-HCl buffer pH 7.1 to a final concentration of 50 mg/mL. The mixture was vigorously homogenized with vortex for 30 s and subsequently centrifuged at 12,000× *g* for 10 min, to obtain an aqueous extract. To the pellet was added 1 mL of methanol, and then it was vortexed and centrifuged at the same conditions. The supernatant was collected and used as methanolic extract.

### 4.4. Antibacterial Assay

The antibacterial activity of extracts was determined by using paper disk diffusion method. Four bacterial strains were utilized as organism test: *Bacillus subtilis* ATCC 6633, *Salmonella typhimurium* ATCC 14021, *Corynebacterium fimi* NTCS 7547, and *Listeria monocytogenes* ATCC 6477. These bacteria were cultivated in 10 mL of LB medium, at 37 °C, for 24 h. Then, 100 µL of bacterial culture was mixed with 25 mL of BHI agar medium (Liofilchem, Roseto Degli Abruzzi, Italy)) at 46 °C and then poured into a Petri dish. Paper discs were treated with 25 µL of extract and were left to dry for two hours. Paper discs (6 mm) impregnated with methanolic (99.8%, Vetec Química Fina, Rio de Janeiro, Brazil) extract were stored at 70 °C, to allow methanol evaporation. The discs were placed on the agar surface and incubated at 37 °C for 24 h.

### 4.5. Volatile Organic Compounds Extraction

The volatile organic compounds were extracted by simultaneous hydrodistillation–extraction. A Likens and Nickerson apparatus was used with *n*-pentane as organic solvent. This process was carried out for 2 h with 3 g of cyanobacterial wet biomass diluted in 250 mL of distillated water.

### 4.6. Gas Chromatography/Mass Spectrometry Analysis (GC/MS)

The volatile organic compounds were analyzed by GC/MS (EI, 70 eV) on a Shimadzu QP 2010 plus instrument (Shimadzu Corporation, Tokyo, Japan) equipped with an Rtx-5MS silica capillary column (30 m × 0.25 mm; 0.25 μm film thickness, Restek Corporation, Bellefonte, PA, USA). Helium was used as carrier gas, adjusted to a linear velocity of 1 mL/min (measured at 100 °C). The column temperature was from 60 to 240 °C, with an increase at a rate of 3 °C/min. The injection type used was splitless (2 mL of a 1:1000 hexane solution) with of 250 °C. The temperature of the ion source and connection parts was maintained at 200 °C. Volatile constituents were determined by comparison of mass fragmentation patterns and molecular weight to authentic standards found in the NIST (National Institute of Standards and Technology,) mass spectral library (Gaithersburg, MD, USA). The retention index was calculated for each compound, using a homologous series of *n*-alkanes (C8–C32, Sigma-Aldrich, Milwaukee, WI, USA) [87].

### 4.7. Statistical Analysis

The antibacterial experiments were performed in triplicate and expressed as means ± standard deviation. Analysis of variance was conducted by Tukey test, following one-way ANOVA; differences at *p* < 0.05 were considered statistically significant, using the software GraphPad 6.0 (GraphPad Software, San Diego, CA, USA, https://www.graphpad.com/).

## 5. Conclusions

The work revealed five volatile organic compounds in *Synechococcus* sp. GFB01. The volatile profile analysis showed the presence of compounds that have never been described for this phylum: 6-pentadecanol and octadecyl acetate. Both are well described in the literature due to their biotechnological potential. However, their contribution to the environment and physiological role has not yet been studied for this group of microorganisms. There is also little information about the genes involved in its biosynthesis. Further studies based on genomics can be used to gain a better understanding about the biosynthetic pathway for the production of these compounds, since its genome sequence is available in NCBI, under accession number NZ_LFEK00000000.1. The studied strain was capable of inhibiting *Salmonella typhimurium* ATCC 14021 growth. By changing phosphate and nitrate concentrations in the medium, it was possible to observe different levels of activity, but not significant. An exception was observed for extract obtained from cells cultivated in medium solely enriched with nitrate, where the activity was abolished.

## Figures and Tables

**Table 1:** Volatile organic compounds of *Synechococcus* sp. strain GFB01.

Compound ^a^	RI Calculated ^b^	RI Literature ^c^	Relative Abundance (%)
Pentadecane	1507	1500	3.75
Hexadecane	1609	1600	2.21
6-Pentadecanol	1685	1675	1.01
Heptadecane	1707	1700	81.32
Octadecyl acetate	2222	2209	11.71

^a^ Compound identified, ^b^ retention index experimentally obtained, and ^c^ retention index from literature.
